# Spatial and Temporal Patterns in Macrofaunal Diversity Components Relative to Sea Floor Landscape Structure

**DOI:** 10.1371/journal.pone.0065823

**Published:** 2013-06-12

**Authors:** Roman N. Zajac, Joseph M. Vozarik, Brittney R. Gibbons

**Affiliations:** 1 Department of Biology and Environmental Science, University of New Haven, West Haven, Connecticut, United States of America; 2 Millstone Environmental Laboratory, Millstone Power Station, Waterford, Connecticut, United States of America; Université du Québec à Rimouski, Canada

## Abstract

We examined temporal changes in macrofaunal α- and β-diversity over several spatial scales (within patches, among patches, across landscapes and across regions) in Long Island Sound on the northeast USA coast. Regional ε-diversity was estimated at 144 taxa, however γ-diversity fluctuated over time as did α- and β-diversity components. Based on additive partitioning, patch- and region-scale β-diversity components generally had the highest contributions to γ-diversity; lower percentages were found at within-patch and landscape scales. Multiplicative diversity partitioning indicated highest species turnover at within- and among patch scales. For all partition results, within-patch and patch-scale β-diversity increased sharply when hypoxia impacted benthic communities. Spatial variation in diversity components can be attributed to the collection of different patch types at varying spatial scales and their associated habitats across the benthic landscapes, as well as gradients in depth and other estuarine-scale characteristics. Temporal variation in diversity components across spatial scales may be related to seasonal changes in habitat heterogeneity, species population dynamics, and seasonal disturbances. Rare species were significant and temporally consistent components of macrofaunal diversity patterns over different spatial scales. Our findings agree with other marine and terrestrial studies that show diversity components vary significantly over different spatial scales and the importance of habitat/landscape heterogeneity in supporting diversity. However, our results indicate that the relative contributions of scale-specific β-diversity components can also change significantly over time. Thus, studies of diversity patterns across patches and landscapes based on data collected at one time, or assembled into a single data set from different times, may not capture the full suite of diversity patterns that occur over varying spatial scales and any time-specific determinants of those patterns. Many factors that shape and maintain sedimentary communities vary temporally, and appear to play an important role in determining and maintaining macrofaunal diversity over different spatial scales.

## Introduction

Biological diversity is a central focus of ecological research, environmental management and conservation. Assessing patterns of diversity over space and time, and the factors and dynamics that govern and generate those patterns, is critical for understanding the ecology of biodiversity and aiding management and conservation efforts. The patterns and dynamics of biodiversity in oceans and estuaries environments are becoming better known, but many gaps exist in our understanding [1]–[3], particularly in sedimentary systems [4]–[8], which comprise the most extensive sea floor environments. There is a relatively long history of diversity studies in coastal and deep sea sedimentary environments [9], generating hypotheses on how diversity is generated and maintained [10]–[12], discussions of global diversity patterns [13], [14] and insights as to the importance of sedimentary biodiversity to marine ecosystem functioning [6], [15]. Although spatial patterns of soft-sediment biodiversity have been addressed to varying extent [9], our knowledge of how different aspects of benthic diversity vary over sea floor landscapes at varying scales in relation to benthic patch structure is rudimentary. Advances in sea floor mapping and related benthic studies [16] now provide the ability to conduct spatially explicit studies of sea floor biodiversity across different spatial scales in relation to the structure of benthic landscapes (hereafter referred to as “benthoscapes”), and the processes that shape benthic biodiversity. Understanding how benthic biodiversity is related to benthoscape structure at different spatial scales, and how t temporal environmental variations may affect such relationships, provides a necessary framework to parse the processes determining the patterns. This study focuses on how macrofaunal diversity components, α, β and γ [17], vary over time across different spatial scales of benthoscape structure in a large estuarine system, using both additive and multiplicative approaches for partitioning diversity We also assess patterns of species rarity across benthoscape structure. Biodiversity in estuaries is at high risk due to impacts from concentrated and growing levels of human development and activity [1], and acquiring a better knowledge of the patterns and dynamics of estuarine biodiversity is time sensitive as species are potentially being lost and habitats irreversibly altered in these important global environments [18].

Ecological diversity is comprised of several components: α-diversity, the diversity within a specific location (sample); β-diversity, the change or difference in diversity among locations (samples), γ-diversity, the overall diversity across landscapes and ε-diversity, the total diversity of a region [17]. Early studies of macrofaunal benthic diversity largely focused on contrasting patterns of α- and γ-diversity, but over the past decade there has been an increased focus on how diversity components, in particular β-diversity, vary spatially and what factors may be responsible for the patterns found. Gray [4], [19] noted the lack of studies on β-diversity in sedimentary environments and the importance of understanding diversity components relative to threats to marine systems, [18]. Since Gray’s publications on diversity in marine systems, increasing numbers of studies have considered patterns of macrobenthic β-diversity in a variety of sea floor environments. For sedimentary systems, studies have shown that β-diversity can vary among taxonomic groups and can be related to changes in varying sets of environmental characteristics across benthic environments, [20]–[22], but distance effects appear to be mixed [22]–[24]. All of the cited studies and others, e.g. [25]–[27], point to the importance of spatial scale in deciphering the determinants of macrofaunal biodiversity, and how these may change across spatial scales. Many recent studies have also underscored what was generally viewed as typical for macrobenthic communities, and that is the relatively high proportions of rare species [23], [28]. Rare species can make significant contributions to diversity at all spatial scales, and their population ecology and role in benthic community dynamics remain effectively unknown.

Marine benthoscapes, like their terrestrial landscape counterparts, are hierarchically structured mosaics of patches that exhibit both within- and among-patch variation in environmental and biotic characteristics as well variation along environmental and structural gradients that occur across the overall structure and composition of the landscape [29], [30]. Josefson [31] used additive diversity partitioning to assess the relative contributions and patterns of diversity components in macrobenthic communities in a set of hierarchically nested samples in the Kattegat/southwestern Baltic Sea area between Denmark and Sweden. He found that β-diversity at the regional scale made a larger contribution to overall diversity than diversity components at smaller spatial scales, likely driven by changes in species composition due to gradients in salinity. In contrast, there have been more studies of diversity partitioning across the spatial hierarchy of landscape structure in terrestrial, e.g. [32]–[37], freshwater [38], [39] and other types of marine systems [40]–[42], providing insights into how α- and β-diversity vary across spatial scales and how spatial patterns of diversity vary among different taxonomic and functional groups of species. Many of these studies point to the importance of landscape- and region-scale contributions to overall diversity, and the role of environmental heterogeneity across these spatial scales in generating the patterns.

Although habitat mapping is providing new tools, types of information, and interesting approaches for the assessment of benthic habitats and communities [43]–[45], there have been no assessments of how macrofaunal diversity components vary relative to the hierarchical structure of benthoscape patch structure that can be revealed through sea floor mapping. Studies of diversity components in marine sedimentary systems, and in other environments, do not often provide an explicit landscape context as landscape components are usually defined by general patch/habitat types separated over distances that encompass general types of landscapes and the regions within which they are located. An explicit landscape context can provide additional insights into how diversity components change over varying scales of landscape structure. Furthermore, no assessments of temporal changes in marine macrofaunal diversity components over varying spatial scales have been made, and there are few for terrestrial systems as well [37], [46], [47]. Within a region, we can expect that α and β will vary within and among different patches across landscapes and with time, especially in temperate, seasonal environments. As such, all species comprising the ε-diversity of a region may not be present at any one time over the sites sampled and as such γ-diversity will vary as well. The objective of our study addresses how macrofaunal diversity components vary over time across the hierarchical structure of two benthoscapes in a large estuarine system along the eastern North American coast. Our null hypothesis was that macrofaunal scale-specific diversity components would show no significant differences in their contribution to overall diversity, and that there would be no significant variation in their relative contributions over time. A secondary objective was to assess temporal variations in species rarity and relationships to the spatial and temporal trends in diversity components that were found.

## Materials and Methods

### Study Areas, Sea Floor Mapping and Sample Collection

**Figure 1:**
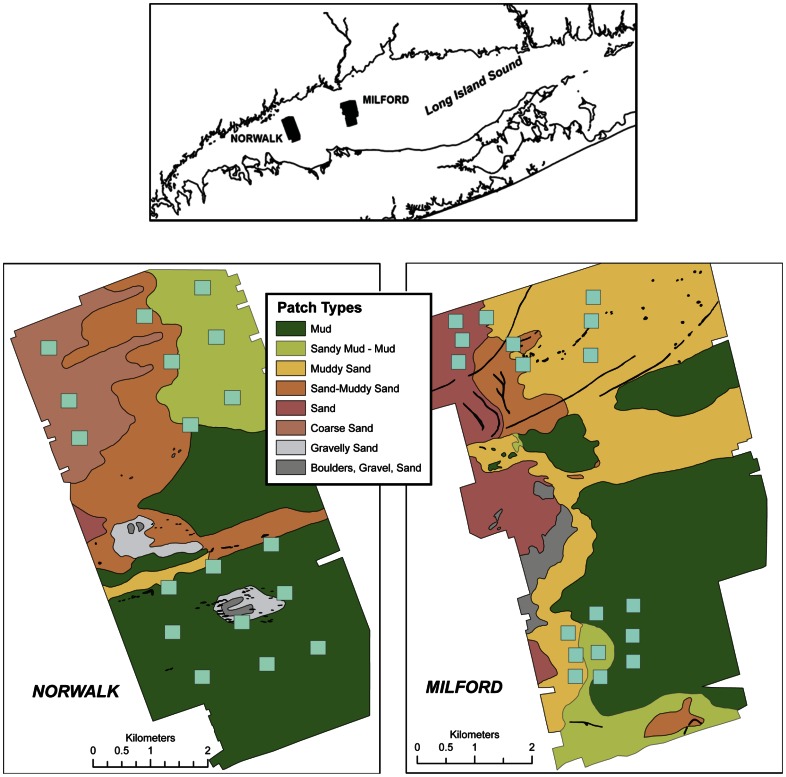
Location and benthoscape patch structure of the two study areas in Long Island Sound, USA. The approximate geographic centers of the study sites are at 41.091772°N, 73.01239°W, and 41.027571°N, 73.282928°W for the Milford and Norwalk sites, respectively. The blue boxes are locations of sampling blocks in different patches.

Data on benthic species composition and abundances were collected in two sea floor benthoscapes in Long Island Sound (LIS), a large estuarine system along the eastern coast of North America (Fig. 1). The benthoscapes were mapped using side scan sonar, one ∼ 8 km south of Milford, CT, and the mouth of the Housatonic River, and the other ∼ 5 km south of the Norwalk Islands. These are referred to as the Milford and Norwalk benthoscapes, respectively. Each was ∼32 km^2^ with water depths ranging from ∼12 to 45 m in each area. Side scan surveys were conducted in November 1993 to develop detailed maps of benthoscape structure in the study areas. Details of side scan operations, side scan mosaic development and interpretation and sediment analyses, as well as high resolution images of the study areas, are provided in Twichell et al. [48], [49]. Based on the side scan mapping, patches of similar acoustic characteristics were identified and a series of spatially nested sampling sites were established in different large-scale patches and along transitions zones in both shallow and deep-water portions of the two benthoscapes (Fig. 1). The differing acoustic characteristics of the large-scale patches were related to different sediment types and habitat features at varying spatial scales and confirmed through subsequent studies [48]–[50]. Briefly, the northern, shallow water portion of the Milford benthoscape is comprised of a large sand patch giving way to a sand/muddy sand transitional patch area, and a large muddy sand patch to the east. In the southern, deeper water area, sands and muddy sand patches give way to a sandy mud – mud transitional patch and a large mud patch to the east. The shallow water, northern portion of the Norwalk benthoscape is comprised of a coarse sand patch in the west, a sand/muddy-sand transitional patch in the center of the mosaic area and a sandy mud/mud patch to the east. The deeper water area is comprised if a muddy sand/sand muddy sand transitional area, a mix of mud and gravely sand/boulders patches and a large mud patch in the most southern portion of the Norwalk benthoscape. LIS experiences significant seasonal environmental fluctuations, including varying degrees of hypoxia and anoxia during summer in its western basins, where the Norwalk benthoscape is located. The area of the Milford benthoscape also experiences hypoxia although not as regularly nor as severely. The maximum period of recruitment for many benthic, sediment dwelling species generally occurs during spring and early summer.

Three 200 m×200 m sampling sites were established within selected patches and transition zones to assess variations in benthic communities across the benthoscapes. The study areas were sampled in April, June, August, and October of 1995, and in June, July, August and October, 1996. Three random, replicate bottom samples were obtained from each site on each sampling date, for a total of 864 bottom samples for the entire project. Samples consisted of a 6 cm diameter×15 cm deep core taken from each grab sample (either a 0.1 m^2^ or 0.05 m^2^ Van Veen grab sampler). The cores were preserved whole in 4% formalin and stained with Rose Bengal and later washed on a 212:m sieve in the laboratory and transferred to 70% ethanol. All organisms were sorted using a dissecting microscope and identified to lowest possible taxon. For each sampling period, species composition from the three core samples within each block was combined and abundances summed to develop the data sets used in the analyses.

### Diversity Analyses

**Figure 2:**
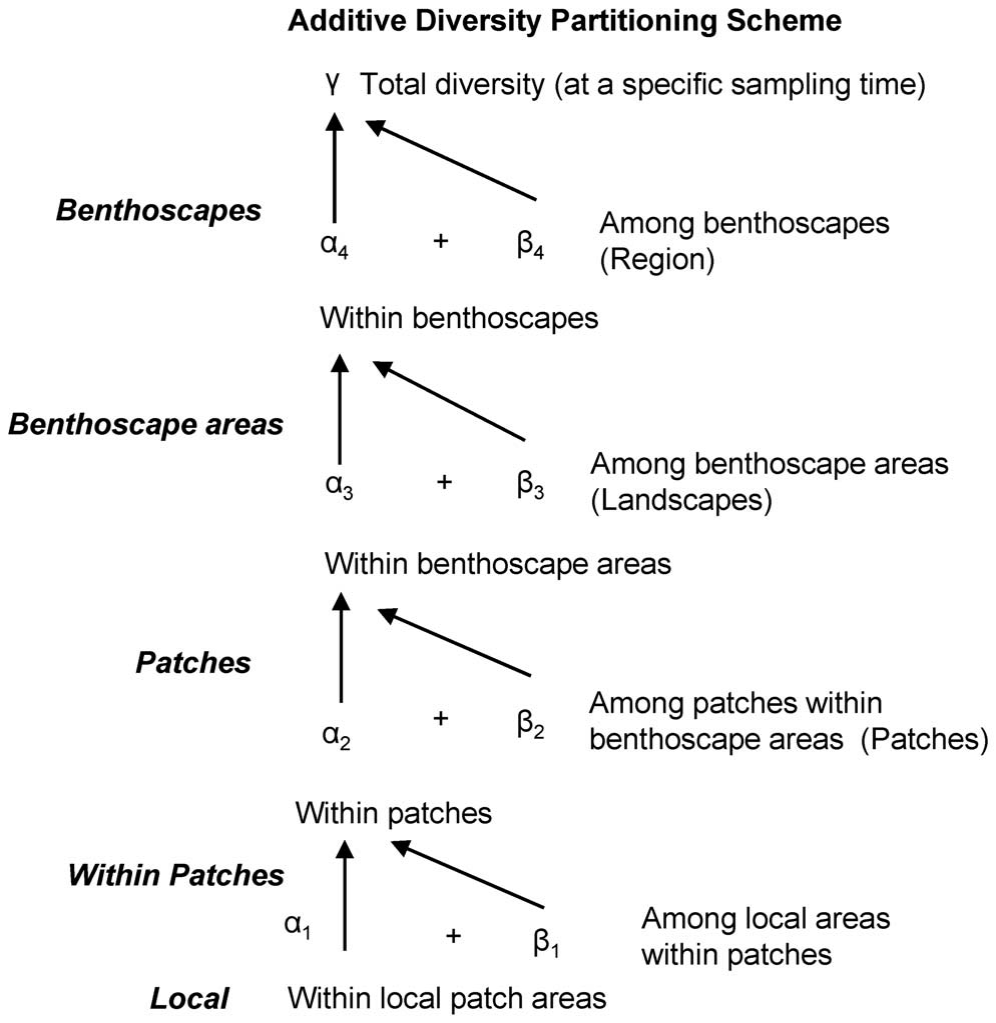
Hierarchical, additive partitioning scheme indicating the spatial scales at which β-diversity components were calculated. Total diversity at a specific sampling time, γ, is calculated as γ = α_1_ (within sampling blocks)+β_1_ (among locations/sampling blocks within patches)+β_2_ (among patches)+β_3_ (among locations with benthoscapes)+β_4_ (among benthoscapes ). The same hierarchical structure was use for multiplicative partitioning, but in this case γ = α_1_×β_1_×β_2_×β_3_×β_4_.

To assess contributions to benthic biodiversity at several spatial scales we employed additive diversity partitioning using Partition 3.0 software [51]. In additive diversity partitioning, γ = total species diversity found in a collection of samples, α = average diversity within the collection of samples, and β = average diversity among samples, so that γ = α+β, and β = γ – α. Across hierarchical sampling levels, with i = 1,2,3,…. m levels, samples at successive nested level i+1 are formed by pooling samples in the level below, i-1. Thus, α_i_ = average diversity found within samples at each level i, and the diversity components are calculated as β_m_ = γ – α_m_ at the highest level and β_i_ = α _i+1_– α _i-1_ for each lower sampling level. The additive partition for diversity is: 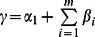. Details on additive diversity partitioning are provided in [33], [52], [53]. For this study, our hierarchical partitioning included local α diversity (within sampling blocks), and β diversity among locations (sampling blocks) within patches, β_1_, among patches, β_2_, among different portions of benthoscapes, β_3_, and among benthoscapes across the region, β_4_ (Fig. 2). The partitioning relates to increasing spatial extent relative to benthoscape structure. Estimates of α diversity are at the scale of the 200×200 m sampling blocks in each patch (based on the summed values of three samples cores as noted above). Estimates of β diversity the among-patch scale, β_2_, represent larger spatial scales than estimates for locations within patches, β_1_. β diversity estimates among portions of benthoscapes,β_3_, e,g, the northern and southern areas of the Milford and Norwalk benthoscapes, represent larger spatial scales than patches, and the regional scales comparing the two benthoscapes, β_4_ represents the largest spatial scale. The β components represent increasing spatial scales of benthoscape structure, but since sampling blocks were not equidistant within specific patches, nor the patches themselves, each spatial scale of the hierarchical partitioning represent a range of actual distances; ∼400 m–1 km for β_1_, ∼800 m –2.4 km for β_2_, ∼4–5 km for β_3_, and ∼20 km for β_4_.

To assess seasonal and year to year changes in diversity components, partitioning was conducted for each sampling period separately. Both weighted (total of number of individuals in a sample as a proportion of the total number of individuals in the data set) and unweighted data were used to partition observed and expected taxonomic richness. The observed diversity components were tested for deviation from randomness based on 999 iterations using individual based randomization to calculate p-values. Diversity component values from each sampling period were grouped to assess differences in average contributions to total diversity across the study period using analysis of variance (ANOVA) or non-parametric Kruskal-Wallis tests. We also calculated multiplicative diversity components of species richness and multiplicative partitions using a range of q-metric diversities [54] to assess the sensitivity of temporal changes in β-diversity components to differences in the diversity partitioning approach used, as well as the influence of rare, q = 0.5, and more abundant species, q = 1.5, on changes in β-diversity across spatial scales; we also used q = 0.999 which yields the common Shannon diversity measure.

Additional analyses were conducted using EstimateS 8.0 software [55] to determine temporal changes in species rarity, including the calculation of singletons (species with only one individual in the pooled samples), doubletons (species with only two individuals in the pooled samples), uniques (species that occur in a only one sample among the samples), duplicates (species that occur in a only two samples among the samples), and the mean number of shared species among sample blocks for each sampling period.

## Results

### General Community Characteristics

A total of 144 benthic taxa were found (from a total sample of >10,000 individuals), providing an estimate of the overall ε-diversity of the region over the two year study period. The species pool was dominated by polychaete annelids (75 taxa), molluscs (27 taxa) and crustaceans (23 taxa), with 19 taxa in other groups. Numerically dominant species included the polychaetes *Mediomastus ambiseta, Ampharete americana, Nephtys incisa, Scalibregma inflatum* and *Cossura longocirrata*, an unidentified oligochaete species and the bivalves *Nucula proxima, Mulinia lateralis* and *Yoldia limatula*. Details of community composition and dynamics are given in [50].

### Diversity Partitioning

**Figure 3:**
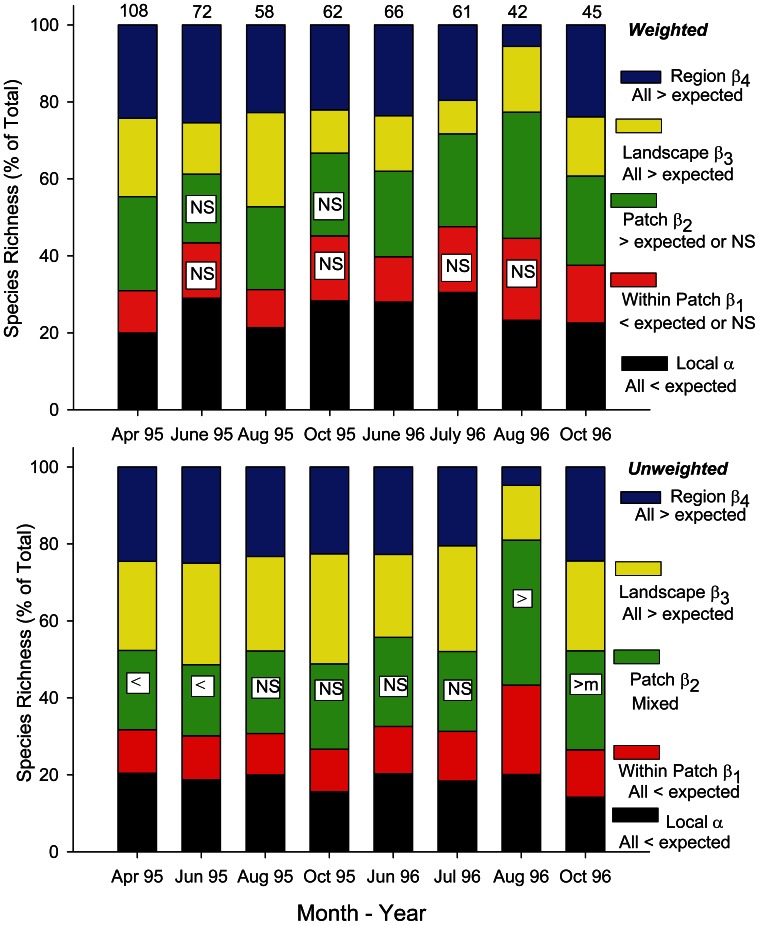
Results of weighted and unweighted additive partitions of species richness across two benthoscapes in Long Island Sound. Randomization test results are given to the right of the figures. “> ” indicates significantly (p<0.05) larger contributions than expected from random to the diversity component at that scale, “< expected indicates significantly smaller contribution; NS indicated not significantly different (p>0.10) from random, m indicates marginally significant (0.05>p<0.10).

**Figure 4:**
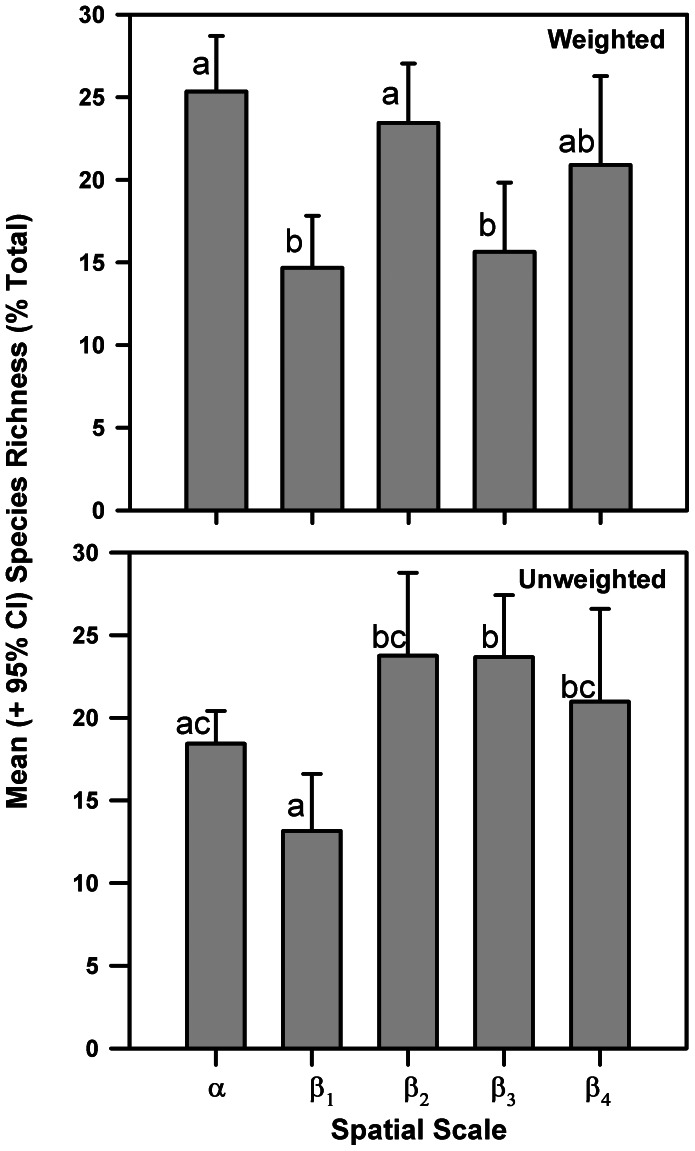
Differences in the mean (+95% confidence interval) temporal contribution of diversity components to species richness in Long Island Sound. Spatial scale associated with each diversity component is given in Fig. 2. Although the mean values are presented for the unweighted partition, a Kruskal-Wallis test was used to test for differences in medians. Lowercase letters show results of post-hoc tests, diversity components sharing the same letters were not significantly different (p<0.05).

The time-specific γ-diversity in each sampling month fell below the estimated ε-diversity for the entire study period, and diversity components based on additive partitioning across different scales of benthoscape structure varied considerably among sampling dates (Fig. 3). Based on sample weighted data, mean local α-diversity was relatively consistent accounting for 19.9–28.9% of total species richness throughout the study period. These levels were all significantly less (p<0.05) than expected from random. The relative contributions to γ-diversity of β-diversity components β_1,_ among locations within patches, β_2_, among patches, and β_3,_ among different portions of benthoscapes, varied considerably over the study period, with no apparent seasonal pattern. β_1_ contributions ranged between 9.8% and 21.2% and were either not significantly different (p>0.05) than expected or significantly less than expected from random. Changes in diversity among patches, β_2_, were not significantly different from random at two sampling periods and otherwise were greater than expected from random, accounting for 17.8% –32.8% of total species richness. Contributions to total species richness among different portions of benthoscapes, β_3_, ranged from 8.7% to 22.7% and were all greater than expected from random. At the regional level, contributions among benthoscapes, β_4_, were relatively consistent over time, ranging from 19.6% to 25.4%, except in August 1996 when it fell to 5.5% (see Discussion). All the β_4_ contributions were significantly greater than expected. The mean contributions of diversity components to total species richness over time (Fig. 4) were significantly different among the spatial scales considered (one way ANOVA, F_4,35_ = 7.69, p<0.001). The highest mean contributions were at the local, α, among patch, β_2_ and among benthoscapes, β_4_, scales, with significantly lower mean contributions at the within-patch, β_1_, and within benthoscapes, β_3,_ scales. Regression analyses indicated no relationships between diversity components and total species richness (R^2^ varied from 0.0 to 0.047, and all slopes were non-significant, with p-values ranging from 0.605 to 1.0).

Diversity partitioning using unweighted data resulted in less variable diversity components over time and somewhat different relative contributions to species richness (Fig. 3). Local mean α-diversity comprised 14% to 20% of species richness over the study period, somewhat less than that found using weighted partitioning. All the observed α values were significantly less than predicted. Contributions of β_1_ diversity, among sites within patches, ranged from 10 to 14%, except in August 1996 when it was 23%, and were all significantly greater than expected. Diversity components at the patch, β_2_, benthoscape, β_3,_ and regional, β_4_, levels made similar contributions to species richness, ranging from 18–28% except in August 1996 when the contribution of β_2_ was 37.6%, and β_3_ and β_4_ accounted for 14.2% and 4.8% of species richness, respectively. In contrast to abundance weighted partitions, observed β_2_ unweighted values were mixed with respect to null models; significantly less than predicted in April and July 1995, not significantly different from expected between August 1995 and July 1996, and either greater than or marginally greater than (0.05>p<0.10) predicted in August and October 1996. All diversity components at larger spatial scales, β_3_ and β_4_, were significantly greater than expected. The relative contributions of unweighted diversity components to total species richness (Fig. 4) were significantly different when pooled over the study period (Kruskal-Wallis test, H = 19.54, p<0.001). Patch, β_2_, benthoscape, β_3_, and region-level, β_4_, components were not significantly different from each other, but all higher than within-patch, β_1_, diversity. Local and within-patch diversity components were not significantly different, and contributions of local α diversity were not different from that found at the patch and region scale.

**Figure 5:**
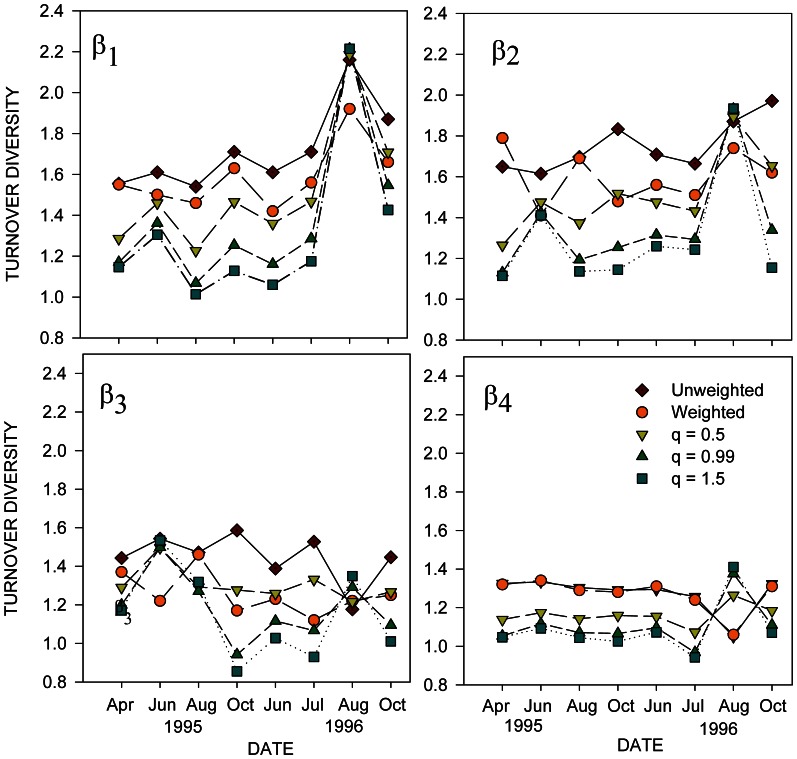
Temporal fluctuations in β turnover diversity at different spatial scales in Long Island Sound, based on multiplicative partitioning. Spatial scales associated with each β-diversity component are given in Fig. 2. Weighted and unweighted refer to species richness.

Patterns of temporal change in multiplicative β components varied over the study period and differed depending on which partitioning approach was employed and the specific β-diversity component considered (Fig. 5). Overall, all q-metric β-diversity components declined with increasing values of q, reflecting the lower emphasis given to rare species as q inreases. However, changes in turnover diversity at each spatial scale over the study period differed among the β components. Beta diversity among locations within patches, β_1_, varied in a similar pattern for all turnover values calculated, fluctuating between April 1995 and July 1996, and then showing a sharp increase in August 1996. The patterns of β-diversity among patches, β_2_, and among different sections of benthoscapes, β_3_, were more variable over time relative to the partitioning approach employed and the value of q, although all q-metric values for β_2_ increased sharply in August 1996 as well. Beta diversity among benthoscapes, β_4_, was generally lower than the other β components, similar among the different q-metrics, and exhibited relatively little variation over time, except for an increase in q-metric β_4_s in August 1996. This is in contrast to the weighted and unweighted values calculated for species richness which declined at that time. Interestingly, multiplicative β-values for the within-patch scale were similar to the patch scale and higher than for landscape and regional scales, in contrast to lower within-patch scale β_1_ values relative to larger spatial scales calculated using additive partitioning of species richness. Randomization tests indicated that most multiplicative β-diversity components were higher than expected for most sampling dates (Table 1). However, β_1_ values were either lower than expected or not significantly different for all q-metric diversity partitions, except in August 1996, and randomization test results were mixed for q = 0.999 and q = 1.5 partitions for most 1996 samples. Testing of general differences among multiplicative β components over the study period, by grouping values for specific β components from each sampling time and applying a Kruskal-Wallis tests, indicated that there were significant differences in species turnover for the weighted (H = 22.583, p<0.001) and unweighted (H = 24.44, p<0.001) species richness partitions, with β_1_, within-patch, and β_2_, patch, contributions significantly greater (p<0.05, Dunn’s post hoc z-test) than landscape and regional scales. For the q-diversity components, there were significant differences for q = 0.05 (H = 20.01, p = 0.002) and q = 0.999 (H = 9.707, p = 0.021), but the median β contributions among spatial scales were not significantly different (H = 5.757, p = 0.123). For q = 0.05 and q = 0.999, within patch and patch level βs were significant higher than for landscape and regional scales (p<0.05, Dunn’s post hoc z-test).

**Table 1:** Results of significance tests of multiplicative β-diversity components from partitioning analyses using individual-based randomizations.

		Weighted	Unweighted	q = 0.5	q = 0.999	q = 1.5
Apr 95	β_1_	+	NS	+	+	+
Apr 95	β_2_	+	+	+	+	+
Apr 95	β_3_	+	+	+	+	+
Apr 95	β_4_	+	+	+	+	+
June 95	β_1_	+	NS	**−**	**−**	**−**
June 95	β_2_	+	+	+	**−**	+
June 95	β_3_	+	+	+	+	+
June 95	β_4_	+	+	+	+	+
Aug 95	β_1_	+	**−**	**−**	**−**	**−**
Aug 95	β_2_	+	+	+	+	+
Aug 95	β_3_	+	+	+	+	+
Aug 95	β_4_	+	+	+	+	+
Oct 95	β_1_	+	**−**	**−**	NS	M+
Oct 95	β_2_	+	+	+	+	+
Oct 95	β_3_	+	+	NS	**−**	NS
Oct 95	β_4_	+	+	+	+	+
June 96	β_1_	+	**−**	**−**	**−**	**−**
June 96	β_2_	+	+	+	+	+
June 96	β_3_	+	+	+	+	M+
June 96	β_4_	+	+	+	+	+
July 96	β_1_	+	+	NS	NS	NS
July 96	β_2_	+	+	+	+	+
July 96	β_3_	+	+	+	NS	**−**
July 96	β_4_	+	+	NS	**−**	**−**
Aug 96	β_1_	+	+	+	+	+
Aug 96	β_2_	+	+	+	+	+
Aug 96	β_3_	+	+	+	+	+
Aug 96	β_4_	+	+	+	+	+
Oct 96	β_1_	+	M+	NS	NS	NS
Oct 96	β_2_	+	+	+	NS	NS
Oct 96	β_3_	+	M+	M+	NS	NS
Oct 96	β_4_	+	+	+	NS	+

+ = significantly (p<0.05) larger than expected from null model, **−** = significantly smaller than expected, M = marginally significant (0.05>p<0.10).

### Rarity

**Figure 6:**
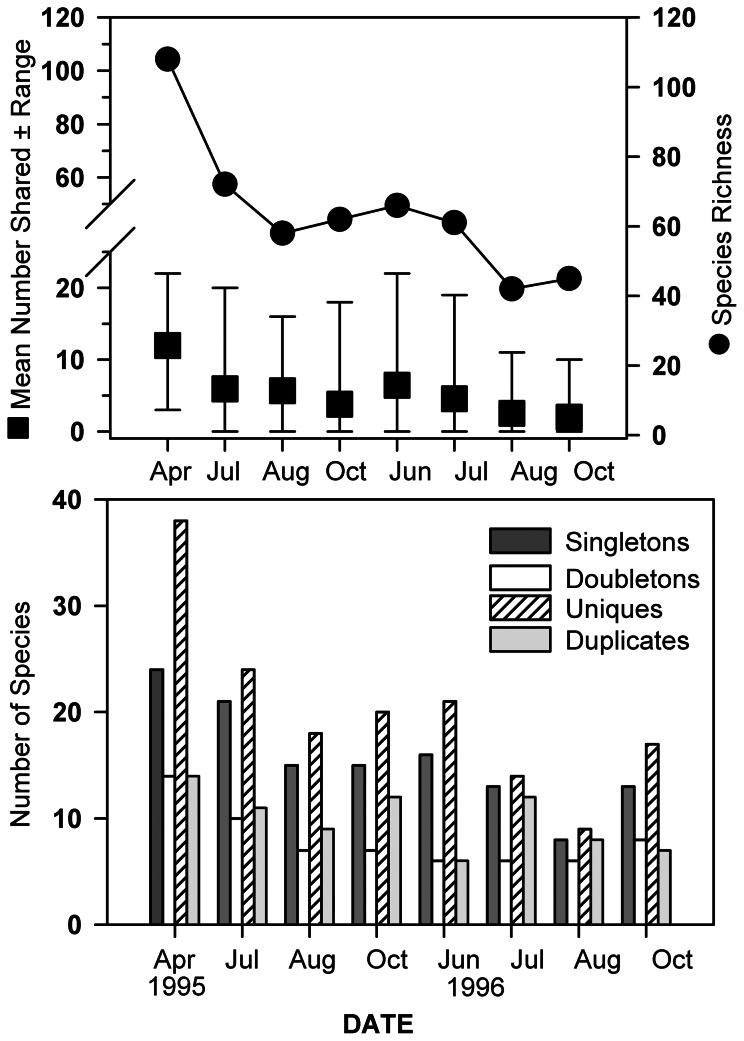
Temporal fluctuations in the mean number of shared taxa (upper) and the number of taxa in several different categories of rarity (lower) in Long Island Sound. Rarity categories are defined in text. The upper graph also shows the fluctuation in the total number of taxa found at each sampling time.

The mean number of shared species among any two locations was generally <10 and tracked fluctuations in overall species richness (Fig. 6). The highest numbers of shared species ranged between ∼ 19 and 25, and there were sampling sites that shared no species on all sampling dates except in April 1995. Several measures of species rarity were calculated for the data set (Fig. 6). The highest numbers of singletons and doubletons (species with only one or two individuals in the pooled samples, respectively) were found in April and July 1995 when species richness was highest and then remained fairly constant over the rest of the study period. The number of uniques (species that occurred at only one site in the pooled samples) was highest in April 1995, and then varied between ∼ 15 and 25 species during the rest of the study period. The number of duplicates (species that occurred at only two sites in the pooled samples) varied between ∼8 and 15 species over the course of the study. 35.4% of the taxa comprising the ε-diversity of the region were found during only one sampling period, and 14.5% were found at two sampling times (Table 2). Only 32 species were found on every sampling date (22.2% of the total number of taxa found). Of the 51 species only found on one sampling date, most were polychaete annelids, followed by gastropods and crustaceans. In terms of life mode/functional attributes, these taxa were predominantly associated with the sediment/water interface, with fewer taxa having a burrowing life mode (Table 2).

**Table 2:** Frequency of rarity of Long Island Sound macrofauna during the 1995–1996 study period, and the taxonomic and functional characteristics of taxa that were not found seven of the eight sampling periods.

Number of times not found:	1	2	3	4	5	6	7
**Number of taxa:**	4	9	9	8	10	21	51
**Polychaetes:**	28		Sed/W Interface:				37
**Gastropods:**	7		Burrowing:				14
**Amphipods:**	4						
**Other Crustaceans:**	4						
**Cnidaria:**	3						
**Bivalves:**	3						
**Echinoderms:**	1						

Sed/W = Sediment/Water.

## Discussion

The diversity components associated with the macrofaunal communities studied in Long Island Sound varied significantly across different spatial scales of seafloor benthoscape structure. Also, their relative contributions to γ-diversity fluctuated over time, with some components exhibiting greater degrees of temporal variation than others. Based on additive species richness partitioning, local α-diversity (i.e. within the 200×200 sampling blocks) tended to be relatively less variable than the β components, and comprised only about ∼18–25% of total species richness. On an overall basis, spatial scales above the local habitat accounted for the greatest proportion, ∼75%, of species richness across the benthoscapes,. The percent contribution of within-patch β_1_ diversity was generally low but variable, and the contribution at the largest spatial scale, β_4,_ among benthic landscapes, was similar throughout the study. However, there were larges shifts in β_1_ and β_4_ in August and October 1996 (see below). The greatest degree of variation was at the among-patches, β_2_ and among-portions of benthoscapes, β_3_, scales for all the diversity partitioning calculation methods employed. Multiplicative β components, including q-metric diversities, exhibited similar degrees of variability in their spatial and temporal patterns, but the relative contributions to overall diversity differed as within-patch scales, β_1_, accounted for higher species turnover relative to β components at larger spatial scales, in contrast to the smaller relative β_1_ contributions among spatial scales indicated by additive partitioning of species richness. The differences between the additive and the q-metric diversities reflect the effects of widespread dominant species and the different units each metric expresses (numbers of species versus species equivalents). Although multiplicative β_1_ components indicated large contributions to γ-diversity, randomization tests often indicated that these were significantly smaller than expected, similar to that found for additive β_1_ components.

Despite some differences in the relative patterns and contributions of the diversity components, on overall basis, certain trends emerge that were generally robust to the partitioning methods employed. Chief among these are the significant contributions β-diversity components make to γ-diversity, and that scale–specific β-diversity components can exhibit significant temporal fluctuations, indicating shifts in the relative contributions/importance of the factors associated with different scales of benthoscape hierarchies. These include both environmental factors and ecological characteristics and dynamics that play out across different spatial and temporal scales. The significant increases in β-diversity at larger spatial scales associated with patch and benthoscapes in this study agree with findings of similar trends in other marine and terrestrial environments. For example, Josefson [31] found larger than expected contributions (>65%) to γ-diversity of benthic macrofauna among regions. Similar to our findings for α-diversities and β_1_ among locations within a specific patch, Josefson [31] also found that α-diversity (within samples) and his β_1_ diversity among samples, were lower than expected. Greater than expected contributions to total diversity at large spatial scales have been found, for example, in coral reef landscapes, for both corals [40], [42] and coral dwelling fish [40], forest insect communities [34], [36], plants in agricultural landscapes [35], macroinvertebrates and fish of lakes and streams [38], [56] and desert mammals [57].

The β-diversity patterns we found point to the importance of benthoscape structure and the associated variation in habitat heterogeneity over different spatial scales and times in shaping the diversity of sedimentary macrofaunal communities. The study areas in Long Island Sound are comprised of large-scale patches of varying sediment type that have small- to meso-scale sedimentary habitat features such as pits, mounds, sandwaves, shell accumulations, and tube mats, that vary both within and among the large-scale patches [50]. Across the two benthoscapes there are also smaller-scale gradients in depth among patches and within patches, and a benthoscape-wide depth gradient from shallow (∼ 5–15 m) to deep (15–35 m) waters. The contributions of β-diversity components to overall diversity relative to this structure indicate that habitat heterogeneity is an important and temporally consistent determinant of this diversity. Considering additive β components, the smallest contribution, ∼14% on average, was found for different locations within specific patches (β_1_), which is not surprising since it is expected that although within-patch habitat features are likely to vary over the extent of a patch, there will be some overall similarity of habitat features relative to other patch types. A spectrum of sedimentary environments comprised the large-scale patches of the LIS benthoscapes, varying from patches of boulders, gravel, and sand to clayey-silty muds (Fig. 1), constituting highly heterogeneous benthoscape elements. Similar sedimentary environments were found in both shallow and deep waters, but depth also adds to overall habitat heterogeneity. The β-diversity components at the patch, β3 and landscape, β4, scales, accounted for ∼40 to 50% (weighted and unweighted additive partitioning, respectively) of total species richness in the region. This likely is related to species accumulating across the different patches of the benthoscapes, and the habitats they provide, as well as responses to depth related habitat characteristics. Regional differences among the two benthoscapes accounted for approximately 22% of species richness, except in August 1996. There are differences in the overall benthoscape structure of the two study areas (Fig. 1), as well as estuarine-scale gradients in environmental and ecological characteristics from the central to the western basins of LIS [58]–[60], where the Milford and Norwalk sites were located, respectively. These differences generate additional habitat differences and niches across the region, allowing for additional species to accumulate at this scale. Collectively, the results from this and the other studies noted above underscore the importance of landscape heterogeneity as a determinant of the ecology of biodiversity in many different types of environments.

Whilst habitat heterogeneity at different spatial scales may have important positive effects on diversity, disturbances can have significant negative effects on sedimentary communities and biodiversity [61], [62], and for some types of disturbances through impacts that reduce habitat heterogeneity [63], [64] All the partition results in this study indicate a sharp shift in the relative contributions of β components in August 1996, which is likely related to the responses of macrofaunal communities to hypoxia/anoxia events. The western portion of LIS, where the Norwalk site was located, is characterized by seasonal hypoxia/anoxia, the extent and severity of which varies from year to year (see: http://www.ct.gov/dep/cwp/view.asp?a=2719&q=325532&depNav_GID=1654 Accessed 2013, January 29). The spatial distribution of low oxygen conditions during periods of hypoxia/anoxia in these portions of LIS is highly variable as well. The Milford site is outside the zone that is generally susceptible to hypoxia/anoxia in the late summer. In mid to late August 1996 there was a significant hypoxia event in the region of Norwalk site, with bottom water dissolved oxygen (DO) levels ranging between 1–2 mgL^−1^. Portions of the Milford site experienced DO levels of 3–3.5 mgL^−1^ at this time. Macrofaunal communities were greatly reduced in abundance and species number at this time and spatially fragmented in terms of remnant communities and species populations [50], [65]. Within-patch, β_1_, and patch, β_2_, diversity components accounted for a greater degree of overall diversity with reductions at the landscape and regional scale. It appears that patches and portions of patches are differentially affected by low DO and this community fragmentation leads to smaller-scale pockets of different species among locations within-patches and among patches across the benthoscapes. Species richness was reduced across both study areas, accounting for reductions in benthoscape, β_3_, and region-level, β_4_, diversity components. This suggests that large-scale disturbances, such as hypoxia in this case, can alter the spatial patterns of β-diversity across sea floor benthoscapes by reducing diversity across the extent of the hypoxic zone and increasing community heterogeneity at smaller spatial scales.

Another important aspect of the spatial and temporal patterns of macrofaunal benthic diversity presented here is the fluctuation in γ-diversity that occurred through the study period. The overall number of taxa found in this study was 144, which can be considered as an estimate of ε diversity [17], the overall diversity of a large region comprised of varied landscapes. However, γ-diversity was consistently lower than ε-diversity, ranging from 108 to 42 taxa. The low γ-diversity found in August and October 1996 can be attributed to the hypoxic event discussed above. The highest γ-diversity was found in April and June 1995, and then remained fairly constant through the middle portion of the study between August 1995 and July 1996. The fluctuations in γ-diversity prior to the August 1996 likely reflect variation in seasonal recruitment across LIS among years, particularly in spring/early summer, and subsequent declines in species richness during summer, fall and winter, which is common in large temperate estuarine systems such as LIS. When environmental conditions allow for strong recruitment years, it is likely that a greater proportion of species comprising ε-diversity will have higher population abundances, mostly in spring and early summer, and be spread across a broader region, and thus more likely to be sampled.

In our study, γ-diversity ranged from 36 to 86 taxa less than the estimated ε-diversity for the region, not counting the sampling periods affected by anoxia in 1996. This difference underscores the contribution of rare species to overall macrofaunal diversity patterns. A large proportion of the taxa collected fell into several categories of rarity. The mean number of shared species among all sampling sites was low, ∼10 taxa, and ranged from 1–28 taxa across the study period. The highest numbers of shared species occurred during spring/early summer recruitment periods, and generally tracked fluctuations in γ-diversity. Consequently, macrobenthic diversity patterns were greatly shaped by spatially and temporally variable occurrences (and disappearances) of the majority of the taxa comprising ε-diversity. Half the taxa were found only once or twice, but the number of uniques, singletons, doubletons and duplicates did not vary greatly among sampling dates, except for the number of uniques at the April 1995 sampling date. Many of the rarest species (found only once during the study) were polychaetes, gastropods and crustaceans that live at sediment/water interface or in the upper few cm of the sediments, such as amphipod crustaceans, and ampharetid, cirratulid and syllid polychaetes. Deeper living, generally burrowing, species may have been undersampled, so there may be some bias in the distribution of life modes among these rare species. Despite this potential bias, the data suggest that macrofauna that occupy habitats associated with top layers of the sediments may be the most likely to vary in their spatial and temporal distribution and, as such, constitute the majority of rare taxa in soft sediment benthoscapes. They may also be most sensitive to variations in habitat heterogeneity associated with the sediment-water interface and differences in these habitat components at the patch and larger spatial scales. Studies have indicated that macrobenthic β-diversity may be positively related to small-scale variations in biogenic habitat heterogeneity [63] and significant variations in β-diversity across benthoscapes may primarily involve taxa that have life modes and life histories that are closely dependent on conditions at the sediment/water interface. The most common taxa found in the study areas, and most widely distributed [50], are mostly either burrowing deposit feeders/omnivores (*Nephtys incisa, Scalibregma inflatum*, *Cossura longocirrata*), or are sedentary or discretely motile but live several cm deep in the sediments (*Nucula proxima, Yoldia limatula*), suggesting that species with these types of life modes are more likely to comprise the majority of shared species in subtidal sedimentary environments such as sampled in this study. It is not known to what extent the rare species found in the benthoscapes we studied are more abundant in other areas of LIS, and this points to the need for spatially extensive surveys over several time periods to obtain an accurate assessment of commonness and rarity. Assessments of system-wide surveys in LIS conducted previous to this study reveal significant spatial variations in macrobenthic community structure across this estuary [66] beyond the largest scale included in our analyses. How patterns of taxonomic rarity and commonness change over such system-wide scales, and how they are related to patterns of diversity, remain a little understood aspect of macrobenthic ecology.

In conclusion, our findings agree with other studies of marine and terrestrial communities that have shown diversity components to vary significantly over different spatial scales and the importance of habitat/landscape heterogeneity at multiple spatial scales in supporting regional diversity. However, our results indicate that the relative contributions of scale specific β-diversity components can also change significantly over time. Thus, studies of diversity patterns across patches and landscapes that are based on data collected at one time, or assembled into a single data set over a short period or from disparate times, may not capture the full suite of diversity patterns that occur over varying spatial scales and any time-specific determinants of those patterns. Many factors that shape and maintain sedimentary macrofaunal communities vary temporally, and appear to play an important role in determining and maintaining macrofaunal diversity over different spatial scales.
